# Clean Milk, Pasteurised Milk, and Sterilised Milk

**Published:** 1908-06-27

**Authors:** 


### Public Health and Hygiene.

### CLEAN MILK, PASTEURISED MILK, AND STERILISED MILK.

Notwithstanding all that has been said and written on the milk question, a discussion recently opened at the Royal Sanitary Institute by Dr. H. R. Kenwood, Chadwick Professor of Hygiene in the University of London, showed that the most diverse views still prevail on this question. Everyone is agreed that babies during the first nine months should be fed exclusively upon their mothers' milk, provided such be available in sufficient quantity and of normal quality. No one disputes that where cow's milk is fed to children it should be pure, fresh, clean, and unsophisticated. Such complete accord in all authoritative opinion would seem to leave no loophole of justification for improper practice or heretical opinion, judged by this standard.

Certainly nothing can justify a counsel that fails to emphasise the all important character of these two propositions. No graver responsibility, having regard to the welfare of the infant, can be undertaken by the medical man who is called upon to advise on the question of substituting artificial for natural feeding. The sorry pretexts that are urged in defence of such substitution show how little appreciated are the terrible risks to the infant incurred in the change. Probably not one-half of the babies over three months of age are fed on their mother's milk, and statistics clearly show that it is among babies artificially fed that the disgracefully high infantile mortality occurs.

Let the campaign of insistence upon the importance of natural feeding of infants be carried on with all the vigour of a united and enthusiastic profession, and there will still remain the fact that large masses of the people will pursue the vicious custom, which in some degree is imposed by uncontrollable conditions. For these and for the children no longer nurslings the question of the milk supply is of paramount importance. Is it possible to secure for our large urban populations a supply of fresh, pure, clean milk? The answers appears to be in the affirmative, with the qualification, " but at a price! " A visit, subsequently to the discussion, made to the Walker-Gordon laboratory at Sudbury, demonstrated not only the fact, but the manner also by which this is secured. There the cows are fed on carefully selected food, housed in unimpeachable byres, milked under aseptic conditions, and the milk cooled, bottled, and distributed under circumstances which leave nothing which scientific prevision can indicate as desirable.

But it was stated that to feed babies with milk thus carefully protected costs something like 20s. a week. It is doubtless an advantage to the rich to be able to secure for their babies such an impeccable milk, but it is admitted that here is no solution of the problem of a public supply of clean milk. From ten to thirty thousand persons die each year in England and Wales from diarrhœa, and by far the greater number of these are children who may be said to have been poisoned by milk-borne contagia.

The chief source of contamination is unquestionably the home where improper storage, domestic carelessness and uncleanliness, the prevalence of the house fly, and other for the time being irremediable conditions, contribute chiefly to this result. It is for these reasons that Professor Kenwood finds himself in agreement with many other sanitarians in thinking that a provisional solution of the question is to be found in the supply of pasteurised milk, distributed in sealed bottles in place of the open cans and dirty jugs in which, for the most part, it is now stored. Professor Kenwood, in opening the discussion, said:

Although the term " pasteurisation " is a term implied to denote a procedure the details of which may vary considerably, let me broadly define it as the exposure of milk (previously cleansed by filtration or centrifugalisation) to a temperature not exceeding 75° C. for a short period; and then rapidly cooling it to a temperature as much below 16° C. as possible. Under such conditions it is possible (*a*) to reduce the micro-organisms which are capable of being cultivated on artificial media to less than 5 per cent. of those which can be cultivated from the original milk; to thereby destroy or inhibit the fermentation bacteria so as to delay the natural souring of the milk some twelve to twenty-four hours, the milk meanwhile keeping perfectly wholesome; (*b*) to destroy the specific organisms of tuberculosis, diphtheria, enteric fever, cholera, and dysentery, and doubtless also, in large measure, those organisms that are causal of zymotic diarrhœa; (*c*) to do away with the necessity for drugging the milk with harmful chemical preservatives. It is, therefore, a valuable measure of protection against the recurrence of those milk-borne epidemics which have figured largely in the epidemiological records of this country; it is a useful means of reducing the grave risks of the infection of tuberculosis in milk; and the evidence is overwhelming that it reduces the suffering and mortality among infants who are artificially fed.

But although it is granted that the use of sterilised and boiled milk occasionally harms a child (for this is testified to by those whose findings are authoritative and beyond dispute), it cannot be in the public interest to discourage the use of such milk in face of the overwhelmingly greater dangers of raw milk. If one sets the danger of sterilised and boiled milk against that of the raw article, the former danger sinks into insignificance by the side of the latter. Those who rail against the use of sterilised milk say, in effect, that rather than one child should suffer from scurvy, some hundreds may be left to die of zymotic diarrhœa, etc.

Judging from the available evidence, it must be extremely rare for *pasteurised milk* to cause infantile scurvy, and it possesses the additional advantages over sterilised milk that its taste is unaltered, the physical changes brought about by pasteurisation are practically nil, and the digestibility is increased (for the fat and lact-albumen are unchanged, the caseinogen is readily and completely coagulable by the rennin ferment into soft clots, and its digestion is not delayed, and there is no precipitation of calcium salts or changes in the phosphates). Pasteurised milk will not, of course, agree with all children, any more than the mother's milk does; but infantile scurvy depends largely upon how long the milk is kept, and pasteurised milk is consumed in a far fresher condition than sterilised milk.

I have spared no pains to inform myself of the case against pasteurised milk from the above standpoint, and I find little, if any, evidence to support the contention that the mere warming of the milk to a temperature not exceeding 75° C. for a few minutes occasions an alteration in its chemical or physical qualities that can materially reduce its nutritional value and induce disturbances of nutrition.

I would reiterate that the pasteurisation of the public milk supply affords a readily available means of bringing about a reduction of infantile sickness and mortality, even if the maximum is conceded to those who seek to discredit it. Recognising as I do the extent of the home-contamination of milk, pasteurised milk supplied in bottles (at 4d. per quart) is, in my opinion, an ideal public supply under present conditions—that is, until we attain to the general practice of elaborate cleanliness both at the farm and the home, and until every milch cow in the country is examined at least four times a year by an independent veterinary expert.

